# Editorial: Clinical implications of targeting lipid metabolism and associated pathways for cancer therapy

**DOI:** 10.3389/fonc.2024.1464240

**Published:** 2024-09-03

**Authors:** Rimsha Munir, Nousheen Zaidi

**Affiliations:** ^1^ Cancer Research Center, University of the Punjab, Lahore, Pakistan; ^2^ Molecular Biology Department, Hormone Lab, Lahore, Pakistan; ^3^ Institute of Microbiology and Molecular Genetics, University of the Punjab, Lahore, Pakistan

**Keywords:** cancer, tumor metabolic microenvironment, cancer therapy, lipid metabolism, clinical implications

Cancer remains one of the most formidable challenges in modern medicine, necessitating continuous innovation in therapeutic strategies. Among the emerging areas of interest, lipid metabolism has garnered significant attention due to its integral role in cancer cell proliferation, survival, and metastasis. This Research Topic of *Frontiers in Oncology* focuses on the clinical implications of targeting lipid metabolism and associated pathways for cancer therapy. By assembling a collection of cutting-edge research and review articles, we aim to highlight the potential of lipid-targeted therapies in improving cancer treatment outcomes.

## The role of lipid metabolism in cancer

Lipid metabolism encompasses a series of biochemical processes involving the synthesis, degradation, and utilization of lipids within cells (1). In cancer, these processes are often dysregulated, facilitating the rapid growth and spread of malignant cells (2). Lipids serve as key components of cellular membranes, signaling molecules, and energy sources, all of which are critical for tumor development and progression (1).

Several studies have demonstrated that cancer cells exhibit increased lipid uptake and *de novo* lipid synthesis (3–5), a phenomenon known as the Warburg effect for lipids (6). Enzymes such as fatty acid synthase (FASN) and acetyl-CoA carboxylase (ACC) are frequently overexpressed in various cancers, making them attractive targets for therapeutic intervention (7–10). The articles in this Research Topic probe into the molecular mechanisms underlying lipid metabolism in cancer and explore innovative strategies to exploit these pathways for therapeutic benefit.

## Microenvironmental factors modulating tumor lipid metabolism: paving the way to better antitumoral therapy

This comprehensive review by Cai et al. elucidates the intricate ways in which the tumor microenvironment influences lipid metabolism in cancer cells. The article highlights the significant roles of hypoxia, acidosis, and stromal cells in modulating lipid metabolic pathways, thereby affecting tumor growth and therapeutic response. Key insights presented in the review include the identification of hypoxia-inducible factors (HIFs) as crucial regulators that enhance lipid uptake and synthesis while reducing lipid utilization in hypoxic tumor regions. These insights are pivotal for developing effective lipid-targeted therapies, as they underscore the complex interplay between the tumor microenvironment and lipid metabolism. Understanding these interactions will be essential for the advancement of novel therapeutic strategies aimed at targeting lipid metabolism in cancer treatment.

## Associations of novel serum lipid index with epithelial ovarian cancer chemoresistance and prognosis

In this study, Li et al. investigate the correlation between serum lipid profiles and chemoresistance in epithelial ovarian cancer (EOC). The findings suggest that specific lipid indices can serve as biomarkers for predicting chemoresistance and prognosis. Major findings include the identification of the HDL-C/LDL-C ratio as an independent protective factor for both progression-free survival (PFS) and overall survival (OS), indicating its potential utility in personalized treatment strategies in EOC.

## Effect of tumor microenvironment on ferroptosis: inhibition or promotion

This article by Xia and Quan explores the dual role of the tumor microenvironment in regulating ferroptosis, a form of lipid peroxidation-driven cell death. The study highlights how factors such as hypoxia and inflammation can either inhibit or promote ferroptosis. Key findings include the identification of hypoxia as a condition that inhibits ferroptosis through the HIF-1α/lncRNA-PMAN pathway, and the role of pro-inflammatory cytokines in modulating ferroptosis, offering insights into how this pathway can be manipulated for therapeutic purposes.

## Orlistat exerts anti-obesity and anti-tumorigenic effects in a transgenic mouse model of endometrial cancer

Xu et al. present data on the anti-tumor effects of orlistat, an FDA-approved weight loss drug, in a mouse model of endometrial cancer. The study demonstrates that orlistat not only reduces obesity but also inhibits tumor growth by targeting lipid metabolism, specifically through the inhibition of FASN. Major findings include significant reductions in body weight and tumor weight in obese mice treated with orlistat, highlighting its potential for repurposing as a cancer therapy.

## Plasma lipidomics profiling in predicting the chemo-immunotherapy response in advanced non-small cell lung cancer

This prospective analysis by Jiang et al. evaluates the potential of lipidomic biomarkers to predict responses to chemo-immunotherapy in patients with advanced non-small cell lung cancer (NSCLC). The study identifies specific lipid signatures associated with treatment response. Key findings include the identification of six lipids as key predictive markers and the development of a clinical combined model with an AUC of 0.87, demonstrating high accuracy in differentiating between disease control and progressive disease. This highlights the utility of lipidomics in guiding personalized cancer therapy.

## Clinical implications and future directions

The research presented in this Research Topic underscores the importance of lipid metabolism in cancer biology and its potential as a therapeutic target. By elucidating the complex interplay between lipid metabolic pathways and the tumor microenvironment, these studies pave the way for novel treatment strategies that could enhance the efficacy of existing therapies and overcome resistance.

**1. Biomarker Development:**

The identification of lipid-based biomarkers, as demonstrated in the studies on ovarian and lung cancers, holds promise for improving patient stratification and treatment personalization. Future research should focus on validating these biomarkers in larger clinical cohorts and exploring their integration into routine clinical practice.

**2. Combination Therapies:**

Targeting lipid metabolism offers a unique opportunity to develop combination therapies. By inhibiting key lipid metabolic enzymes, such as FASN, in conjunction with standard chemotherapy or immunotherapy, it may be possible to enhance therapeutic efficacy and mitigate resistance. The potential synergistic effects of such combinations warrant further investigation in clinical trials.

**3. Dietary Interventions:**

The study on orlistat highlights the potential of dietary interventions and metabolic modulation in cancer therapy. Incorporating dietary strategies that influence lipid metabolism, alongside pharmacological treatments, could offer a holistic approach to cancer management (11). Clinical studies evaluating the impact of dietary modifications on treatment outcomes are needed.

**4. Novel Therapeutic Agents:**

Continued exploration of novel agents targeting lipid metabolism is essential. The development of specific inhibitors for enzymes like ACC, FASN, and others involved in lipid synthesis and uptake could provide new therapeutic avenues. Preclinical and clinical studies should aim to assess the safety and efficacy of these agents in various cancer types.

## Conclusion

The articles featured in this Research Topic of Frontiers in Oncology highlight the critical role of lipid metabolism in cancer and its potential as a therapeutic target. By advancing our understanding of lipid metabolic pathways and their interaction with the tumor microenvironment, these studies contribute to the development of innovative treatment strategies that could significantly improve cancer patient outcomes. We hope this Research Topic inspires further research and clinical translation in the field of lipid-targeted cancer therapy.
